# Supplementary material for: Diagnostic Performance of Digital Breast Tomosynthesis for Breast Suspicious Calcifications From Various Populations: A Comparison With Full-field Digital Mammography
Source: Comput Struct Biotechnol J. 2018 Dec 20;17:82–9. doi: 10.1016/j.csbj.2018.12.004 (PMC6317146; doi:10.1016/j.csbj.2018.12.004)
Supplement: Supplementary file 1 — Supplementary material [file mmc1.docx]

**Diagnostic performance of** **digital breast tomosynthesis for breast suspicious calcifications from various populations: a comparison with full-field digital mammography**

Juntao Li^a^, Hengwei Zhang^a^, Hui Jiang^b^, Xuhui Guo^a^, Yinli Zhang^c^, Dan Qi^d,e^, Jitian Guan^d,e^, Zhenzhen Liu^a^, Erxi Wu^d,e,f,g,h*^, Suxia Luo^i,*^

^a^Department of Breast Surgery, Affiliated Cancer Hospital of Zhengzhou University, Henan Cancer Hospital, Zhengzhou 450008, China;

^b^Department of Radiology, Affiliated Cancer Hospital of Zhengzhou University, Henan Cancer Hospital, Zhengzhou 450008, China;

^c^Department of Rheumatology, the First Affiliated Hospital of Zhengzhou University, Zhengzhou 450052, China;

^d^Department of Neurosurgery, Baylor Scott & White Health, Temple, TX 76508, USA;

^e^Neuroscience Institute, Baylor Scott & White Health, Temple, TX 76502, USA;

^f^Department of Surgery, Texas A&M University College of Medicine, College Station, TX 77807, USA;

^g^Department of Pharmaceutical Sciences, Texas A&M University College of Pharmacy, College Station, TX 77807, USA;

^h^LIVESTRONG Cancer Institutes, Dell Medical School, the University of Texas at Austin, Austin, TX 78712, USA;

^i^Department of Medical Oncology, Affiliated Cancer Hospital of Zhengzhou University, Henan Cancer Hospital, Zhengzhou 450008, China.

**E-mail addresses:** [JuntaoLi@hotmail.com](mailto:JuntaoLi@hotmail.com) (J. Li); [hengwei@medmail.com.cn](mailto:hengwei@medmail.com.cn) (H. Zhang); [jhhappy2014@163.com](mailto:jhhappy2014@163.com) (H. Jiang); [guoxuhui1980@qq.com](mailto:guoxuhui1980@qq.com) (X. Guo); [fcczhangyl@zzu.edu.cn](mailto:fcczhangyl@zzu.edu.cn) (Y. Zhang); [Dan.Qi@BSWHealth.org](mailto:Dan.Qi@BSWHealth.org) (D. Qi); [Jitian.Guan@BSWHealth.org](mailto:Jitian.Guan@BSWHealth.org) (J. Guan) ; [liuzhenzhen73@126.com](mailto:liuzhenzhen73@126.com) (Z. Liu) ; [Erxi.Wu@BSWHealth.org](mailto:Erxi.Wu@BSWHealth.org) (E. Wu); [suxialuo66@126.com](mailto:suxialuo66@126.com) (S. Luo).

***Corresponding authors:**

Suxia Luo, M.D., Department of Medical Oncology, Affiliated Cancer Hospital of Zhengzhou University, Henan Cancer Hospital, 127 Dongming Road, 450008, Zhengzhou, China. Fax: +86 (0371) 65587783, Tel: +86 (0371) 65587783.

E-mail: [suxialuo66@126.com](mailto:suxialuo66@126.com)

Erxi Wu, Ph.D., Neuroscience Institute and Department of Neurosurgery, Baylor Scott & White Health, 5701 Airport Road, Temple, TX 76502, USA. Tel: +1 (254) 7243785.

E-mail: [Erxi.Wu@BSWHealth.org](mailto:Erxi.Wu@BSWHealth.org)

**Highlights:**

1. DBT exhibits higher diagnostic accuracy for benign calcifications compared to FFDM

2. DBT exhibits higher diagnostic accuracy in dense breast cases than FFDM

3. DBT exhibits higher diagnostic accuracy in premenopausal and postmenopausal cases

4. DBT has no advantage in non-dense breasts and malignant calcifications cases

**Abstract:**

The diagnostic performance difference between digital breast tomosynthesis (DBT) and conventional full-field digital mammography (FFDM) for breast suspicious calcifications from various populations is unclear. The objective of this study is to determine whether DBT exhibits the diagnostic advantage for breast suspicious calcifications from various populations compared with FFDM. Three hundred and five patients were enrolled (of which seven patients with bilateral lesions) and 312 breasts images were retrospectively analyzed by three radiologists independently. The postoperative pathology of breast calcifications was the gold standard. Breast cancer was diagnosed utilizing DBT and FFDM with sensitivities of 92.9% and 88.8%, specificities of 87.9% and 75.2%, positive predictive values of 77.8% and 62.1%, negative predictive values of 96.4% and 93.6%, respectively. DBT exhibited significantly higher diagnostic accuracy for benign calcifications compared with FFDM (87.9% *vs* 75.2%), and no advantage in the diagnosis of malignant calcifications. DBT diagnostic accuracy was notably higher than FFDM in premenopausal (88.4% *vs* 78.8%), postmenopausal (90.2% *vs* 77.2%), and dense breast cases (89.4% *vs* 81.9%). There was no significant difference in non-dense breast cases. In our study, DBT exhibited a superior advantage in dense breasts and benign calcifications cases compared to FFDM, while no advantage was observed in non-dense breasts or malignant calcifications cases. Thus, in the breast cancer screening for young women with dense breasts, DBT may be recommended for accurate diagnosis. Our findings may assist the clinicians in applying the optimal techniques for different patients and provide a theoretical basis for the update of breast cancer screening guideline.

**Keywords:** Breast calcification; Digital breast tomosynthesis; Full-field digital mammography

**Abbreviations**

ACR, American College of Radiology; ACS, American Cancer Society; AUC, The area under the ROC curve; BI-RADS, The Breast Imaging Reporting and Data System; CC, Craniocaudal position; DBT, Digital breast tomosynthesis; DCIS, Ductal carcinoma *in* *situ*; FFDM, Full-field digital mammography; MLO, Mediolateral oblique position; ROC, The receiver operating characteristic.

**1. Introduction**

Breast calcification is a common manifestation in the breast cancer screening. A majority of calcifications are benign, while some may indicate the presence of early-stage breast cancer [[1](#_ENREF_1)]. It is quite challenging to distinguish malignant calcifications from benign using current imaging techniques. The Breast Imaging Reporting and Data System (BI-RADS) of the American College of Radiology (ACR) clearly describes the morphology, distribution, and categories of breast calcifications, including typical benign calcifications and calcifications with suspicious morphology [[2](#_ENREF_2), [3](#_ENREF_3)]. Some suspicious calcifications, especially clustered sand-like micro-calcifications often indicate a malignant disease such as ductal carcinoma *in situ* (DCIS) [[4](#_ENREF_4)]. Hence, the identification of suspicious calcifications is crucial.

Conventional full-field digital mammography (FFDM) is a 2-dimensional imaging technique which is widely used to screen for early breast cancer and diagnose breast lesions [[5](#_ENREF_5)]. However, it has some inevitable limitations because of its inability to accurately distinguish suspicious lesions from the adjacent overlapping tissue [[6](#_ENREF_6)]. For instance, FFDM has been faulted for its high false-positive rate and low sensitivity, especially in women with dense tissue [[7](#_ENREF_7)].

Digital breast tomosynthesis (DBT) is a 3-dimensional imaging technique developed to overcome some of the limitations of conventional FFDM [[5](#_ENREF_5)], and has been increasingly employed in breast cancer screening and assessment [[8-10](#_ENREF_8)]. It removes the overlapping of breast tissue which can mask breast abnormalities, potentially raise sensitivity for breast cancers, and decrease the false-positive rate [[11](#_ENREF_11)]. DBT also improves preoperative breast cancer staging in dense breasts patients significantly [[12](#_ENREF_12)]. Meanwhile, DBT shows higher detection rate and diagnostic accuracy for both benign and malignant masses, with better sensitivity and specificity and lower recall rates [[13](#_ENREF_13)]. Our previous study also showed that DBT had a significant advantage over FFDM in the accuracy of diagnosis of breast cancer [[14](#_ENREF_14)], especially in the diagnosis of breast mass-like lesions. However, this technology has not been widely used in China.

Multiple breast imaging studies have implemented the combined procedure of FFDM and DBT [[15-17](#_ENREF_15)]. The combination of FFDM and DBT improved breast cancer accuracy, decreased the false-negative rate, and increased the sensitivity as compared to using only FFDM. The primary limitations of FFDM plus DBT for screening and clinical diagnosis are the increase of interpretation time [[18](#_ENREF_18)] and radiation dose [[19](#_ENREF_19)]. To break through these limitations, the synthetic 2-dimensional (2D) images are reconstructed from the information acquired during a DBT data acquisition procedure [[19](#_ENREF_19)]. DBT plus synthetic 2D imaging increases cancer detection rates and the image reading times compared with FFDM, with comparable recall rates [[20](#_ENREF_20)]. While for the evaluation of microcalcifications, the diagnostic performances of synthetic 2D imaging and FFDM were not significantly different, whether performed with DBT or alone [[21](#_ENREF_21)]. TOMMY Trial found that synthetic 2D imaging plus DBT demonstrated similar performance to that of standard FFDM plus DBT, while the addition of DBT increased the sensitivity of FFDM in patients with dense breasts and the specificity of FFDM for all subgroups [[22](#_ENREF_22)]. Some other studies estimated the 3D positions of the microcalcifications in each of the clusters and reconstructed the clusters as ellipsoids by utilizing multiple projections and the geometry of DBT, as well as demonstrated a possible way of 3D shape analysis by utilizing DBT to make the diagnosis more accurate [[23](#_ENREF_23)]. These studies indicated that DBT might possess a potential benefit as a stand-alone modality in the screening and diagnosis, other than an adjunct of 2D imaging.

However, the detectability of stand-alone DBT for the breast microcalcifications is still controversial. Some studies demonstrated that DBT enabled the detection and characterization of microcalcifications with no significant differences from FFDM [[24](#_ENREF_24), [25](#_ENREF_25)]. Kopans *et al.* found that the clarity of DBT images in 92% cases was equal to or better than that of conventional mammography, and was judged to be better in almost half cases than conventional mammography [[26](#_ENREF_26)]. In contrast, some other studies demonstrated that FFDM appears to be more sensitive than DBT for the detection of calcification [[27](#_ENREF_27), [28](#_ENREF_28)]. To our knowledge, few studies have focused on the comparison of DBT and FFDM for breast suspicious calcifications with different breast densities and menopausal status. The aim of our analyses was to determine whether DBT exhibits the diagnostic advantage for breast suspicious calcifications from various populations compared with FFDM and assist the clinicians in applying appropriate method for different patients.

**2. Materials and Methods**

**2.1. Patients**

This retrospective study was approved by the Ethics Committee of the Affiliated Cancer Hospital of Zhengzhou University (Henan Cancer Hospital) and the patients provided written informed consent for the surgical biopsy and imaging. The study was conducted between 03/2015 and 03/2018. Study participation was offered to women who met the following recruited criteria: (a) participants who underwent FFDM and DBT examinations; and (b) calcification was found through either FFDM or DBT and was classified into category 4A or above according to BI-RADS of ACR [[2](#_ENREF_2)]; (c) the lesions were finally confirmed by histopathology through surgical biopsy in our hospital. The exclusion criteria were the following: (a) mammography indicated typical benign calcifications [[2](#_ENREF_2), [3](#_ENREF_3)]; (b) pregnant or lactating women; (c) participants who underwent breast surgery or breast treatment. We included a final cohort of 312 breast suspicious calcified lesions from 305 women (of which seven cases with bilateral lesions) (age range 31–72 years; mean age 48.7 years).

**2.2. Image Acquisition**

The patients underwent FFDM and DBT imaging of both breasts in the craniocaudal (CC) and mediolateral oblique (MLO) positions using a standard DBT system (Selenia Dimensions 5000, Hologic, USA) before surgery. The specifications of this machine are as follows: detector pixel size 3328×4096; resolution 7.1 lp/mm; pixel pitch 70 μm. Using the standard imaging phantom in the combo mode (DBT plus FFDM), the average glandular radiation doses for FFDM, DBT, and combo mode in a single view are approximately 1.25, 1.65, and 2.90 mGy, respectively. DBT examination was performed immediately after FFDM in the same compression mode (combo mode) using automatic exposure control by the same designated technician. For DBT, while the x-ray tube rotated through an arc of -7.5 to +7.5 degrees, a series of low-dose 1 mm-thick 2D images were obtained while the breast was compressed in the fixed position. These images were reconstructed into a series of 1 mm-thick slices using the filtered back projection technique automatically.

**2.3. Image Analysis**

Three radiologists (Hengwei Zhang, Hui Jiang, and Xuhui Guo) with 7–18 years of breast image experience participated in the image analysis. All readers had participated in prior reader studies of interpreting tomosynthesis examinations and had undergone training in the interpretation of DBT images. A standard hanging protocol was used to display both the FFDM and DBT images. Image interpretation was performed per breast, not per patient. The retrospective double-blind method was used. Each radiologist first evaluated the FFDM images randomly while blinded to the DBT images and the patient’s clinical information, and assigned a BI-RADS category. Similarly, they evaluated the DBT images randomly while blinded to the FFDM images and the patient’s clinical information. To minimize the learning and memory bias, they evaluated the DBT images and assigned the BI-RADS category with a one-month interval at least [[24](#_ENREF_24), [29](#_ENREF_29)]. When the assigned BI-RADS categories within the same imaging modality were inconsistent among the radiologists, a consensus was reached through discussion [[15](#_ENREF_15)].

Breast density was rated according to ACR BI-RADS categories as follows [[2](#_ENREF_2)]: (a) almost entirely fatty-ACR1; (b) scattered fibro-glandular-ACR2; (c) heterogeneously dense-ACR3; and (d) extremely dense-ACR4. In this study, the first two types (ACR1-2) were classified as non-dense glandular and the latter two types (ACR3-4) as dense glandular.

Breast suspicious calcifications were characterized utilizing BI-RADS categories [[2](#_ENREF_2)] from 2 to 5 to assess the probability of malignancy, as: 2, benign; 3, probably benign (0-2% malignant), initial short-interval (6 months) follow-up suggested; 4, suspicious abnormality, malignancy further stratified as 4A, >2% but ≤10%; 4B, >10% but ≤50%; and 4C, >50% but <95%; 5, highly suggestive (≥95% malignant), appropriate action should be taken. The categories 0 (requiring additional imaging evaluation or prior mammograms for comparison), 1 (negative), and 6 (known biopsy-proven malignancy) were not applicable, because this was a retrospective study. BI-RADS categories 2, 3, 4A were identified as negative, while categories 4B, 4C and 5 were identified as positive [[30](#_ENREF_30)]. The histopathology findings from surgery were used as the gold standard for the diagnosis of breast cancer. Category 4A means low suspicion of malignancy according to ACR BI-RADS [[2](#_ENREF_2)]. Though histological diagnosis is necessary, the possibility of a benign lesion is much larger than the possibility of a malignant lesion. Therefore, we regarded BI-RADS 4A as negative in our analysis to avoid the dilution of the criteria of malignancy by a high number of benign lesions.

**2.4. Statistical Analysis**

Statistical analysis was performed using GraphPad Prism software (GraphPad Software Inc. CA, USA，version 6.02) and MedCalc software (MedCalc Software bvba, version 15.2.2). The overall comparison of clinical performance was derived from the differences between the mean area under the receiver operating characteristic (ROC) curve. The χ^2^ test was used to determine differences in the final BI-RADS categories of breast suspicious calcifications based on the images taken with the two methods. Fisher’s exact test was used to compare the sensitivity, specificity and to ascertain the difference in performance according to patient breast density and menopausal status. All statistical tests were 2-sided; a *p-*value<0.05 was considered to be statistically significant.

**3. Results**

**3.1. Clinicopathological Characteristics**

A total of 305 patients with 312 separate sites of suspicious calcifications were included in the final study population. Nighty-eight (31%) sites of suspicious calcifications were proved malignant and 214 (69%) were benign. The final histologic results of malignant calcifications were invasive ductal carcinoma, ductal carcinoma *in situ*, mucinous carcinoma, and apocrine carcinoma. The benign lesions were hyperplasia with calcification, adenopathy, atypical hyperplasia, and cystic hyperplasia (**Table 1**). Regarding breast density, 226 (73%) breasts were dense (ACR3-4), whereas 86 (27%) were non-dense (ACR1-2). Regarding menstrual status, 187 (61%) patients were premenopausal (two patients with bilateral breast [lesion](javascript:;)s), whereas 118 (39%) patients were postmenopausal (five patients with bilateral breast [lesion](javascript:;)s). The average glandular doses for a single view were 1.68±0.67 mGy (range, 0.63-4.12 mGy) for FFDM, 2.04±0.57 mGy (range, 1.08-4.32 mGy) for DBT, and 3.72±0.98 mGy (range, 1.71-6.47 mGy) for combo mode.

**Table 1.** Pathological Types of Malignant and Benign Cases

| Pathology Type | *n* (%) |
| --- | --- |
| Malignant | 98 |
| Invasive ductal carcinoma | 43 (44) |
| Ductal carcinoma *in situ* | 51 (52) |
| Mucinous carcinoma | 3 (3) |
| Apocrine carcinoma | 1 (1) |
| Benign | 214 |
| Hyperplasia with calcification | 97 (45) |
| Adenopathy | 86 (40) |
| Atypical hyperplasia | 12 (6) |
| Cystic hyperplasia | 19 (9) |

**3.2. Calcification Characterization**

Breast suspicious calcifications were characterized using BI-RADS categories from 2 to 5 to assess the probability of malignancy. The different BI-RADS categories of DBT and FFDM for suspicious calcifications are shown in **Table 2**. The difference of BI-RADS categories distribution for benign calcifications between DBT and FFDM was statistically significant, while no significant difference was observed in malignant calcifications. In benign group, FFDM classified more cases into BI-RADS 4B, 4C and 5 which were identified as positive (44 cases vs 25 cases).

**Table 2.** BI-RADS Distribution of Suspicious Calcifications Detected by DBT and FFDM Modes

| BI-RADS | 2 | 3 | 4A | 4B | 4C | 5 | χ2 | *p* Value |
| --- | --- | --- | --- | --- | --- | --- | --- | --- |
| Malignant |  |  |  |  |  |  |  |  |
| DBT | 0 | 3 | 5 | 39 | 46 | 5 | 7.263 | 0.2018 |
| FFDM | 3 | 7 | 3 | 44 | 39 | 2 |  |  |
| Benign |  |  |  |  |  |  |  |  |
| DBT | 44 | 86 | 59 | 17 | 7 | 1 | 27.183 | 0.0001 |
| FFDM | 39 | 44 | 77 | 40 | 12 | 2 |  |  |

Abbreviations: DBT, digital breast tomosynthesis; FFDM, full-field digital mammography; BI-RADS, Breast Imaging Reporting and Data System.

**3.3. Diagnostic Accuracy of DBT and FFDM for Suspicious Calcifications**

According to histopathology results, the prevalence of breast cancer was 31.4% (98/312). Utilizing BI-RADS category 4B as a cut-off for diagnosing breast cancer in both DBT and FFDM, DBT diagnostic accuracy for benign calcifications was significantly higher than FFDM (87.9% *vs* 75.2%, χ2=10.494, *p*=0.0012). The diagnostic accuracy of DBT for malignant calcifications was slightly higher than FFDM, but the difference was not statistically significant (92.9% *vs* 88.8%, χ2=0.551, *p*=0.4581). The total diagnostic accuracy of DBT was higher than that of FFDM, with a statistically significant difference (89.4% *vs* 79.5%, χ2=10.986, *p*=0.0009). The areas under the ROC curves (AUC) were 0.904 (95% CI 0.865-0.934) for DBT and 0.820 (95% CI 0.773-0.861) for FFDM, and the difference was statistically significant (Z=5.502, *p*<0.0001) (**Table 3**).

Breast cancer was diagnosed utilizing DBT and FFDM with sensitivities of 92.9% (91/98) and 88.8% (87/98), specificities of 87.9% (188/214) and 75.2% (161/214), positive predictive values (PPV) of 77.8% (91/117) and 62.1% (87/140), negative predictive values (NPV) of 96.4% (188/195) and 93.6% (161/172), positive likelihood ratio (+LR) of 7.64 and 3.58, negative likelihood ratio (-LR) of 0.08 and 0.15, respectively. The specificity of DBT was higher than that of FFDM, with a statistically significant difference (87.9% *vs* 75.2%, χ2=25.04, *p*<0.0001). No significant difference was observed in the sensitivity between DBT and FFDM (92.9% *vs* 88.8%, χ2=2.25, *p*=0.1250). There were no adverse events.

**Table 3.** Diagnostic Accuracy of DBT and FFDM for Breast Calcifications

| Accuracy | Malignant | Benign | Total | AUC (95％CI) |
| --- | --- | --- | --- | --- |
| DBT | 92.9%  (91/98) | 87.9%  (188/214) | 89.4%  (279/312) | 0.904  (0.865- 0.934) |
| FFDM | 88.8%  (87/98) | 75.2%  (161/214) | 79.5%  (248/312) | 0.820  (0.773-0.861) |
| χ2 | 0.551 | 10.494 | 10.986 | 5.502 (Z) |
| *p* Value | 0.4581 | 0.0012 | 0.0009 | <0.0001 |

Abbreviations: DBT, digital breast tomosynthesis; FFDM, full-field digital mammography; AUC, the area under the ROC curve.


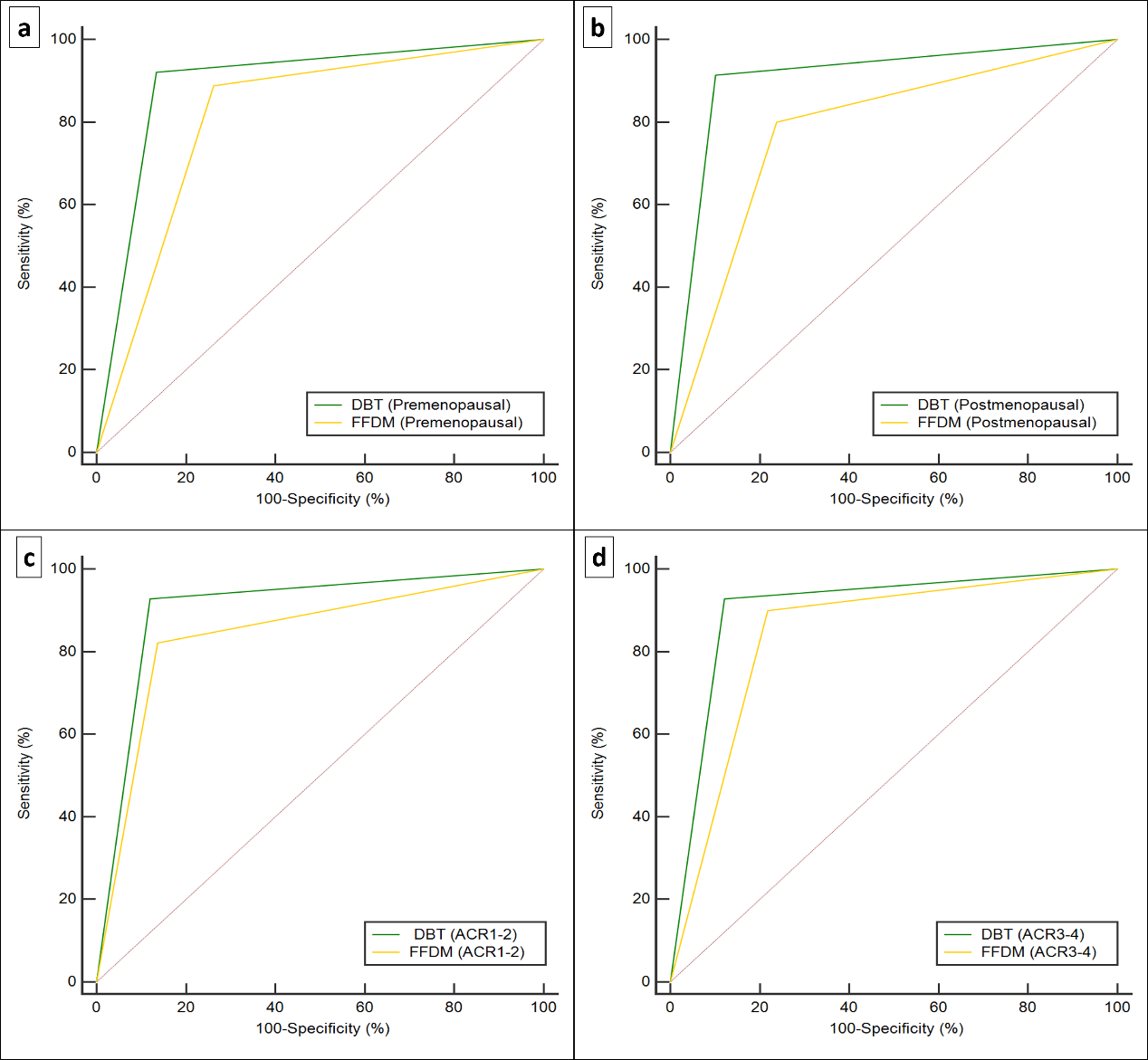


**Fig. 1.** Performance curves of pooled data for all readers.

Data are receiver operating characteristic (ROC) Curves for DBT (green line) versus FFDM (orange line) in the diagnostic accuracy in premenopausal (a), postmenopausal (b), dense breast (ACR1-2) (c) and non-dense breast (ACR3-4) (d) cases.

Abbreviations: DBT, digital breast tomosynthesis; FFDM, full-field digital mammography; ACR, American College of Radiology; ROC, the receiver operating characteristic curve.

**3.4. Comparisons of the Diagnostic Accuracy of DBT and FFDM in Patients with Different Menopausal Status and Breast Densities**

The ROC curves of DBT and FFDM in premenopausal, postmenopausal, dense breast and non-dense breast cases are shown in **Fig. 1** and **Table 4**.

**Table 4.** Comparisons of Diagnostic Accuracy of DBT and FFDM with Different Menstrual Status and Breast Densities

| Characteristics | Accuracy | χ2 | *p* Value | FNR | AUC (95％CI) |
| --- | --- | --- | --- | --- | --- |
| Premenopausal |  |  |  |  |  |
| DBT | 88.4%  (167/189) | 5.576 | 0.0182 | 11.6%  (22/189) | 0.893  (0.840-0.933) |
| FFDM | 78.8%  (149/189) |  |  | 21.2%  (40/189) | 0.813  (0.751-0.866) |
| Postmenopausal |  |  |  |  |  |
| DBT | 90.2%  (111/123) | 6.717 | 0.0095 | 9.8%  (12/123) | 0.906  (0.840-0.951) |
| FFDM | 77.2%  (95/123) |  |  | 22.8%  (28/123) | 0.781  (0.697-0.850) |
| ACR1-2 |  |  |  |  |  |
| DBT | 89.5%  (77/86) | 0.469 | 0.4934 | 10.5%  (9/86) | 0.904  (0.821-0.957) |
| FFDM | 84.9%  (73/86) |  |  | 15.1%  (13/86) | 0.842  (0.747-0.912) |
| ACR3-4 |  |  |  |  |  |
| DBT | 89.4%  (202/226) | 4.600 | 0.0320 | 10.6%  (24/226) | 0.903  (0.857-0.939) |
| FFDM | 81.9%  (185/226) |  |  | 18.1%  (41/226) | 0.841  (0.787-0.886) |

Abbreviations: DBT, digital breast tomosynthesis; FFDM, full-field digital mammography; ACR, American College of Radiology; FNR, False-Negative Rate; AUC, the areas under the ROC curves.

Of all 305 patients, 187 patients were premenopausal, whereas 118 patients were postmenopausal. The diagnostic accuracy of DBT and FFDM with different menopausal status is shown in **Table 4**. The diagnostic accuracy of DBT in premenopausal patients was higher than that of FFDM, with a statistically significant difference (88.4% *vs* 78.8%, χ2=5.576, *p*=0.0182). In postmenopausal patients, the diagnostic accuracy of DBT and FFDM were 90.2% and 77.2% respectively, and the difference was also statistically significant (χ2=6.717, *p*=0.0095). Of all 305 patients with 312 calcification clusters, 226 breasts were classified as dense (ACR3-4) and the remaining 86 as non-dense (ACR1-2). The diagnostic accuracy of DBT in dense breast cases was notably higher than that of FFDM (89.4% *vs* 81.9%, χ2=4.600, *p*=0.0320). In non-dense breast cases, the diagnostic accuracy of DBT was slightly higher than that of FFDM, but the difference was not statistically significant (89.5% *vs* 84.9%, χ2=0.469, *p*=0.4934).

**4. Discussion**

In the present study, we evaluated the diagnostic accuracy of 3D DBT relative to that of 2D mammography FFDM for breast suspicious calcifications, found that DBT could increase the sensitivity and specificity of the diagnosis of breast suspicious calcifications, which is quite helpful for the identification of benign calcifications, especially in young people with higher gland density. The diagnostic sensitivity of DBT and FFDM on breast calcifications were 92.9% and 88.8%; specificity were 87.9% and 75.2%, respectively, and the differences were statistically significant. DBT significantly increased the diagnostic accuracy of total cases, from 79.5% for FFDM to 89.4% for DBT. The diagnostic accuracy difference between DBT and FFDM was significant for benign cases. Whereas, for malignant cases, a significant difference was not [observe](javascript:;)d. The malignancy rates reported in the previous literatures ranged from 10% (8/78) to 39% (41/105) [[31-33](#_ENREF_31)], which was consistent with our result. In our study, 98 (31%) sites of suspicious calcifications were proved malignant and 214 (69%) were benign. Our study did not include similar numbers of benign and malignant calcifications. It is possible that the less numbers of malignant calcification cases disturbed the difference between DBT and FFDM.

Our results seem to contradict several previous studies. Clauser *et al.* found that the diagnostic performance of DBT was as good as that of FFDM; however, a notable inter-reader difference was observed [[24](#_ENREF_24)]. They concluded DBT enabled the detection and characterization of micro-calcifications with no notable differences from FFDM. Even some investigators agreed that FFDM appeared to be slightly more sensitive than DBT for the detection of calcifications [[27](#_ENREF_27)]. The inconsistency between their findings and ours may be due to some factors. First, these studies included fewer cases of calcifications, which adds the possibility of inaccuracy. Second, Clauser’s study focused on comparing the differences between different readers and found high inter-reader variability in the use of the descriptors [[24](#_ENREF_24)], maybe this high inter-reader variability disturbed his research results. However, a few different studies indicated that inter-reader variability did not affect the accuracy, sensitivities and specificities between different methods for predicting the probability of malignancy [[21](#_ENREF_21), [29](#_ENREF_29)]. Whether the inter-reader variability influences the diagnosis is still controversial. Dibble *et al.* found that DBT decreased inter-reader variability, increased the readers’ confidence, and improved sensitivity in detecting breast architectural distortion [[29](#_ENREF_29)]. In clinical application, accurately identifying findings from the mammography is mainly dependent on the reader’s experience. In our study, we did not evaluate the inter-reader variability and the reading times. However, to decrease inter-reader variability as far as possible and guarantee a relatively accurate BI-RADS category, three experienced radiologists participated in prior training in the interpretation of DBT images. A consistent diagnosis among the three readers was employed in our study to decrease the bias of inter-reader variability and guarantee the diagnosis more accurate, which was consistent with Ohashi’s study [[15](#_ENREF_15)].

In our study, all the DBT images were acquired using the narrow-angle (15°/15 projections) modality. The detectability of DBT is dependent on the tomographic scan angle, the number of projections, the radiation dose, and the reconstruction methods. In the wide-angle modality, owing to the greater tissue scanned by X-rays and the decreased dose per projection, the signals received by the detector lower and the relative noise increases, which may reduce the visibility of small structures (including micro-calcifications) [[28](#_ENREF_28)]. Maybe the use of wide-angle (50°/25 projections) DBT also can explain the results of Clauser’s study [[24](#_ENREF_24)].

Due to the pixels binning in DBT, the pixel pitch of DBT is larger than 2D-mammography, which makes DBT images look less sharp than FFDM images. Furthermore, the tube movement in DBT and the relative noise increase of each projection in wide-angle DBT may contribute to the geometric blurring [[28](#_ENREF_28)]. Nevertheless, mild blurring of DBT images can’t mask its advantage in breast screening and diagnosis [[8-13](#_ENREF_8)]. According to the Mammography Quality Standards Act (MQSA) limit (a breast dose restriction of 3 mGy per acquisition) [[34](#_ENREF_34)], slightly increasing the radiation dose of DBT may improve the blurring of DBT images. For the machine in our study, using the standard imaging phantom in the combo mode (DBT plus FFDM), the average glandular radiation doses for FFDM, DBT and combo mode in a single view are approximately 1.25, 1.65 and 2.90 mGy, respectively. Every patient recruited in our research underwent FFDM and DBT imaging in combo mode. Although the average glandular radiation dose for combo mode doubled the dose for FFDM, the overall dose to the breast was within the MQSA limit. Hence, there is no need to worry that the use of this FDA-approved technique would be associated with any harm to the patients [[35](#_ENREF_35)]. Osteras *et al.* investigated the average glandular dose in paired FFDM and DBT acquisitions in a population-based screening program (including 3819 women) and found that the mean dose for FFDM, DBT and Combo was 1.72, 2.09 and 3.81 mGy, respectively [[36](#_ENREF_36)]. Our study observed similar results. In the clinical practice, some parameters of the machine (including the tube loading and voltage) are determined by the automatic exposure control according to the features of the breast (compressed breast thickness and glandular composition), which make the actual breast doses vary between acquisitions.

Meanwhile, we characterized the suspicious calcifications using BI-RADS categories from two to five to assess the probability of malignancy. The difference of BI-RADS categories distribution for benign calcifications between DBT and FFDM was statistically significant, while the difference for malignant was not and thus both DBT and FFDM are alternative detection methods for malignant cases. Our results suggest that the accuracy of DBT in classifying benign calcifications is significantly higher than that of FFDM, and DBT classify more benign calcifications into BI-RADS 3 and 4A categories, [probably](javascript:;) [because](javascript:;) DBT relatively reduced the influence of overlapping tissues and radiologists are able to better assess the 3D character of a lesion in various planes [[37](#_ENREF_37), [38](#_ENREF_38)]. Furthermore, we observed that, for malignant lesions, both DBT and FFDM classified most malignant calcifications into BI-RADS 4B, 4C and 5 categories, which avoided delay in diagnosing the disease. In patients with benign calcifications, FFDM classified a significant proportion of patients into BI-RADS 4B category (**Fig. 2**), which may lead to unnecessary tests or even biopsies. This further proves that DBT has the advantage of avoiding unnecessary biopsies in patients with benign conditions manifest as micro-calcifications. This observation regarding benign lesions is consistent with Tagliafico’s study [[25](#_ENREF_25)].

In premenopausal, postmenopausal and dense breast cases, DBT diagnostic accuracy was higher than FFDM, with a statistically significant difference. But there was no significant difference in non-dense breast cases. This [indicate](javascript:;)s the diagnostic advantage of DBT in premenopausal, postmenopausal and dense breast populations. In Asian countries, including China, the female mammary gland is small and dense [[39](#_ENREF_39)]. Compared to FFDM, DBT exhibits potential benefits in patients with dense breasts, such as reducing recall rates in screening mammography [[6](#_ENREF_6)], improving preoperative cancer staging [[12](#_ENREF_12)], improving cancer detection and mammographic sensitivity [[17](#_ENREF_17)] by eliminating overlapping tissues, which can reduce false-positive rates and the number of biopsies.

According to the updated breast cancer screening guidelines [[40](#_ENREF_40)], the American Cancer Society (ACS) currently recommends that women should undergo regular screening mammography starting at age 45 years. For women younger than 45 years, some may choose to be screened based on cancer individual risk factors, particularly those with family history [[41](#_ENREF_41)]. The traditional mammography is not very useful in younger women owing to the dense breast tissue, making it harder to see potential cancers. DBT may be a better choice for screening in younger women, especially with micro-calcifications.

This study had several limitations. First, we mainly recruited patients who underwent surgical biopsy for suspicious calcifications (BI-RADS 4A category or above) and agreed with this study. The patient who was diagnosed with suspicious calcification BI-RADS 2 or 3 category by both DBT and FFDM were excluded from our study. The bias of the population selection was inevitable. Second, this was a retrospective study and the patients were not randomized, which may not completely represent the clinical problem. Third, we reviewed the images and the pathological results mainly from our workstation and three readers were all from our hospital, which may not be consistent with a multicenter design. Nonetheless, the results of this study still provide clues not only in the diagnostic performance but also in the clinical operation, such as hookwire localization of breast suspicious calcification.

Preoperative hookwire localization is an essential tool in the surgical management of non-palpable breast lesions, especially suspicious micro-calcifications [[42](#_ENREF_42)]. It is generally performed under 2D mammography guidance at the discretion of the radiologist. Then the computer calculates the skin entry site and the path to the lesion. Owing to the scattered and stereoscopic distribution of micro-calcifications, and overlapping images of 2D mammography, inaccurate localization occurs frequently, which requires a relocation. DBT images can reveal the spatial distribution of calcifications [[23](#_ENREF_23)], which may make the hookwire localization more accurate. Further studies on how to improve this technique is still needed.


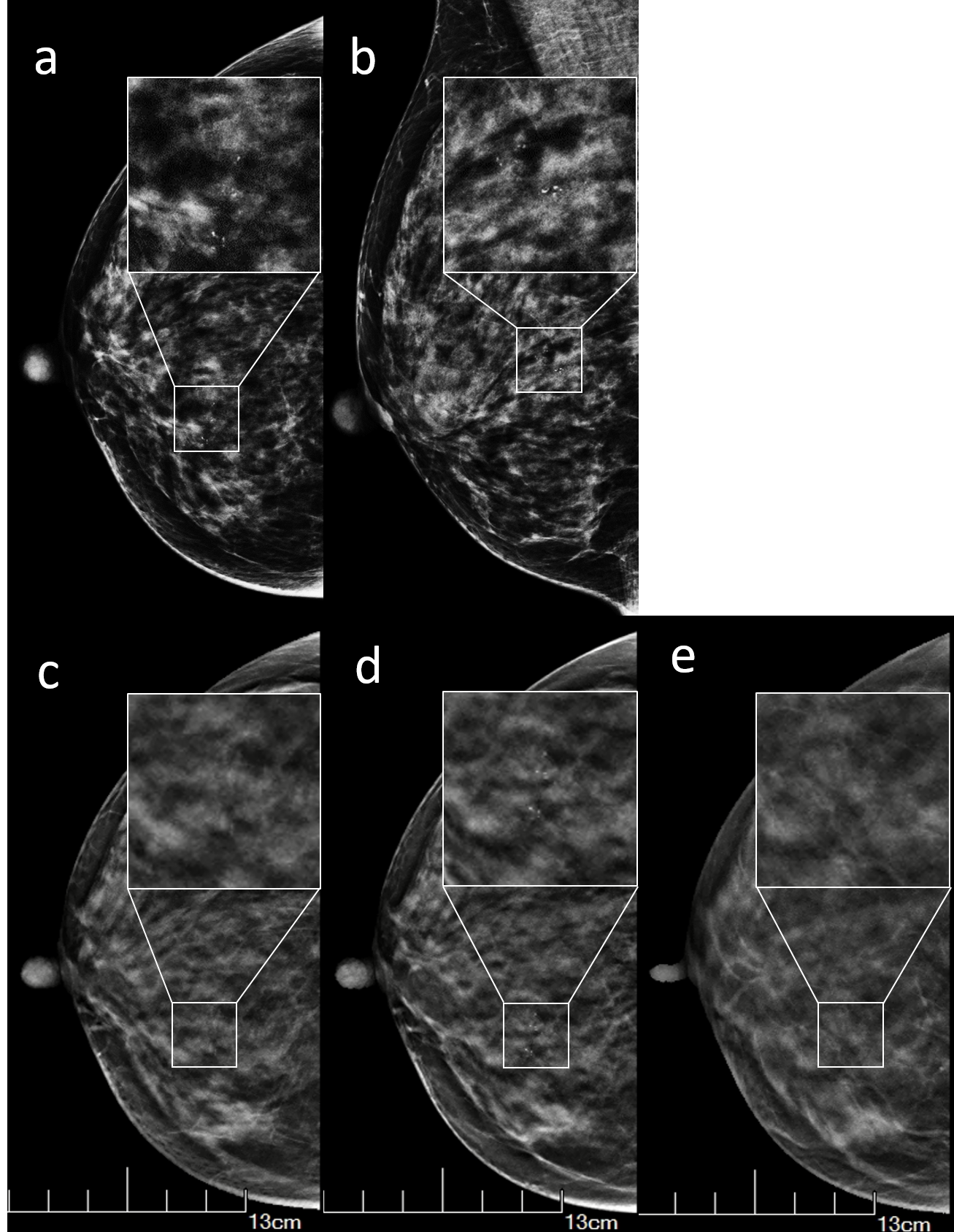


**Fig. 2.** FFDM and DBT images of a 47-year-old woman with adenopathy.

FFDM with CC (a) and MLO (b) view of the right breast reveals irregular micro-calcification (white box) in the lower inner quadrant and classifies it into BI-RADS 4B. DBT with CC views (c-e) dynamically reveal the spatial distribution of calcification more detailedly in the same patient and classifies it into BI-RADS 4A.

Abbreviations: DBT, digital breast tomosynthesis; FFDM, full-field digital mammography; CC, craniocaudal position; MLO, mediolateral oblique position; BI-RADS, Breast Imaging Reporting and Data System.

**5. Conclusions**

From our data, compared with the conventional FFDM, DBT increased the sensitivity and specificity of the diagnosis of breast suspicious calcifications, which was beneficial for the identification of benign calcifications, especially in the young women with dense breasts. DBT exhibited a superior advantage in dense breasts and benign calcifications cases, while no advantage was observed in non-dense breasts or malignant calcifications cases. In the breast cancer screening for young women with dense breasts, DBT may be recommended for accurate diagnosis. Thus, our findings may assist the clinicians in applying the optimal techniques for different patients and provide a theoretical basis for the update of breast cancer screening guideline.

**Declarations**

***Competing interests***

There is no competing interest to claim in this study.

***Ethical approval***

This retrospective study in this paper was approved by the Ethics Committee of the Affiliated Cancer Hospital of Zhengzhou University (Henan Cancer Hospital) and the patients provided written informed consent for the surgical biopsy and imaging. The study was conducted between 03/2015 and 03/2018.

***Fund***

This study was supported by Henan Provincial Medical Science and Technology Research Project, China (No.201702250).

**Acknowledgments**

We thank Xiaomi Fu, BSM, from Department of Breast Surgery, Affiliated Cancer Hospital of Zhengzhou University, for her contribution to data collection.

**References**

1. Keranen AK, Haapea M, Rissanen T. Ultrasonography as a Guiding Method in Breast Micro-Calcification Vacuum-Assisted Biopsies. Ultraschall Med. 2016; 37(5):497-502.

2. Sickles EA, D’Orsi CJ, Bassett LW, Appleton CM, Berg WA, Burnside ES. Acr bi-rads® mammography. ACR BI-RADS® atlas, breast imaging reporting and data system. 2013; 5.

3. Spak DA, Plaxco JS, Santiago L, Dryden MJ, Dogan BE. BI-RADS((R)) fifth edition: A summary of changes. Diagn Interv Imaging. 2017; 98(3):179-90.

4. Tse GM, Tan PH, Cheung HS, Chu WC, Lam WW. Intermediate to highly suspicious calcification in breast lesions: a radio-pathologic correlation. Breast Cancer Res Treat. 2008; 110(1):1-7.

5. Johnson MM. Full-Field Digital Mammography and Digital Breast Tomosynthesis. Radiol Technol. 2017; 88(3):299M-319M.

6. McDonald ES, McCarthy AM, Akhtar AL, Synnestvedt MB, Schnall M, Conant EF. Baseline Screening Mammography: Performance of Full-Field Digital Mammography Versus Digital Breast Tomosynthesis. AJR Am J Roentgenol. 2015; 205(5):1143-8.

7. Carney PA, Miglioretti DL, Yankaskas BC, Kerlikowske K, Rosenberg R, Rutter CM, Geller BM, Abraham LA, Taplin SH, Dignan M, et al. Individual and combined effects of age, breast density, and hormone replacement therapy use on the accuracy of screening mammography. Ann Intern Med. 2003; 138(3):168-75.

8. Ciatto S, Houssami N, Bernardi D, Caumo F, Pellegrini M, Brunelli S, Tuttobene P, Bricolo P, Fanto C, Valentini M, et al. Integration of 3D digital mammography with tomosynthesis for population breast-cancer screening (STORM): a prospective comparison study. Lancet Oncol. 2013; 14(7):583-9.

9. Friedewald SM, Rafferty EA, Rose SL, Durand MA, Plecha DM, Greenberg JS, Hayes MK, Copit DS, Carlson KL, Cink TM, et al. Breast cancer screening using tomosynthesis in combination with digital mammography. JAMA. 2014; 311(24):2499-507.

10. Conant EF, Beaber EF, Sprague BL, Herschorn SD, Weaver DL, Onega T, Tosteson AN, McCarthy AM, Poplack SP, Haas JS, et al. Breast cancer screening using tomosynthesis in combination with digital mammography compared to digital mammography alone: a cohort study within the PROSPR consortium. Breast Cancer Res Treat. 2016; 156(1):109-16.

11. Baker JA, Lo JY. Breast tomosynthesis: state-of-the-art and review of the literature. Acad Radiol. 2011; 18(10):1298-310.

12. Krammer J, Stepniewski K, Kaiser CG, Brade J, Riffel P, Schoenberg SO, Wasser K. Value of Additional Digital Breast Tomosynthesis for Preoperative Staging of Breast Cancer in Dense Breasts. Anticancer Res. 2017; 37(9):5255-61.

13. Bian T, Lin Q, Cui C, Li L, Qi C, Fei J, Su X. Digital Breast Tomosynthesis: A New Diagnostic Method for Mass-Like Lesions in Dense Breasts. Breast J. 2016; 22(5):535-40.

14. Zhang HW, Li JT, Lu MH, Qin L, Li LF, Cui SD, Liu H. [The breast cancer diagnosis accuracy of the digital breast tomosynthesis technique]. Zhonghua Yi Xue Za Zhi. 2017; 97(18):1387-90.

15. Ohashi R, Nagao M, Nakamura I, Okamoto T, Sakai S. Improvement in diagnostic performance of breast cancer: comparison between conventional digital mammography alone and conventional mammography plus digital breast tomosynthesis. Breast Cancer. 2018; 25(5):590-6.

16. Choi WJ, Kim HH, Lee SY, Chae EY, Shin HJ, Cha JH, Son BH, Ahn SH, Choi YW. A comparison between digital breast tomosynthesis and full-field digital mammography for the detection of breast cancers. Breast Cancer. 2016; 23(6):886-92.

17. Seo M, Chang JM, Kim SA, Kim WH, Lim JH, Lee SH, Bae MS, Koo HR, Cho N, Moon WK. Addition of Digital Breast Tomosynthesis to Full-Field Digital Mammography in the Diagnostic Setting: Additional Value and Cancer Detectability. J Breast Cancer. 2016; 19(4):438-46.

18. Zuley ML, Bandos AI, Abrams GS, Cohen C, Hakim CM, Sumkin JH, Drescher J, Rockette HE, Gur D. Time to diagnosis and performance levels during repeat interpretations of digital breast tomosynthesis: preliminary observations. Acad Radiol. 2010; 17(4):450-5.

19. Svahn TM, Houssami N, Sechopoulos I, Mattsson S. Review of radiation dose estimates in digital breast tomosynthesis relative to those in two-view full-field digital mammography. Breast. 2015; 24(2):93-9.

20. Caumo F, Zorzi M, Brunelli S, Romanucci G, Rella R, Cugola L, Bricolo P, Fedato C, Montemezzi S, Houssami N. Digital Breast Tomosynthesis with Synthesized Two-Dimensional Images versus Full-Field Digital Mammography for Population Screening: Outcomes from the Verona Screening Program. Radiology. 2018; 287(1):37-46.

21. Choi JS, Han BK, Ko EY, Kim GR, Ko ES, Park KW. Comparison of synthetic and digital mammography with digital breast tomosynthesis or alone for the detection and classification of microcalcifications. Eur Radiol. 2018.

22. Gilbert FJ, Tucker L, Gillan MG, Willsher P, Cooke J, Duncan KA, Michell MJ, Dobson HM, Lim YY, Suaris T, et al. Accuracy of Digital Breast Tomosynthesis for Depicting Breast Cancer Subgroups in a UK Retrospective Reading Study (TOMMY Trial). Radiology. 2015; 277(3):697-706.

23. Ho CP, Tromans C, Schnabel JA, Brady M. Classification of clusters of microcalcifications in digital breast tomosynthesis. Conf Proc IEEE Eng Med Biol Soc. 2010; 2010:3166-9.

24. Clauser P, Nagl G, Helbich TH, Pinker-Domenig K, Weber M, Kapetas P, Bernathova M, Baltzer PAT. Diagnostic performance of digital breast tomosynthesis with a wide scan angle compared to full-field digital mammography for the detection and characterization of microcalcifications. Eur J Radiol. 2016; 85(12):2161-8.

25. Tagliafico A, Mariscotti G, Durando M, Stevanin C, Tagliafico G, Martino L, Bignotti B, Calabrese M, Houssami N. Characterisation of microcalcification clusters on 2D digital mammography (FFDM) and digital breast tomosynthesis (DBT): does DBT underestimate microcalcification clusters? Results of a multicentre study. Eur Radiol. 2015; 25(1):9-14.

26. Kopans D, Gavenonis S, Halpern E, Moore R. Calcifications in the breast and digital breast tomosynthesis. Breast J. 2011; 17(6):638-44.

27. Spangler ML, Zuley ML, Sumkin JH, Abrams G, Ganott MA, Hakim C, Perrin R, Chough DM, Shah R, Gur D. Detection and classification of calcifications on digital breast tomosynthesis and 2D digital mammography: a comparison. AJR Am J Roentgenol. 2011; 196(2):320-4.

28. Hadjipanteli A, Elangovan P, Mackenzie A, Looney PT, Wells K, Dance DR, Young KC. The effect of system geometry and dose on the threshold detectable calcification diameter in 2D-mammography and digital breast tomosynthesis. Phys Med Biol. 2017; 62(3):858-77.

29. Dibble EH, Lourenco AP, Baird GL, Ward RC, Maynard AS, Mainiero MB. Comparison of digital mammography and digital breast tomosynthesis in the detection of architectural distortion. Eur Radiol. 2018; 28(1):3-10.

30. Su X, Lin Q, Cui C, Xu W, Wei Z, Fei J, Li L. Non-calcified ductal carcinoma in situ of the breast: comparison of diagnostic accuracy of digital breast tomosynthesis, digital mammography, and ultrasonography. Breast Cancer. 2017; 24(4):562-70.

31. Ferreira V, Etchebehere E, Bevilacqua JLB, de Barros N. Suspicious amorphous microcalcifications detected on full-field digital mammography: correlation with histopathology. Radiol Bras. 2018; 51(2):87-94.

32. Esen G, Tutar B, Uras C, Calay Z, Ince U, Tutar O. Vacuum-assisted stereotactic breast biopsy in the diagnosis and management of suspicious microcalcifications. Diagn Interv Radiol. 2016; 22(4):326-33.

33. Scaperrotta G, Ferranti C, Capalbo E, Paolini B, Marchesini M, Suman L, Folini C, Mariani L, Panizza P. Performance and role of the breast lesion excision system (BLES) in small clusters of suspicious microcalcifications. Eur J Radiol. 2016; 85(1):143-9.

34. **Radiation-Emitting Products: Mammography Quality Standards Act and Program** [<https://www.fda.gov/Radiation-EmittingProducts/MammographyQualityStandardsActandProgram/default.htm>]

35. Yaffe MJ, Mainprize JG. Risk of radiation-induced breast cancer from mammographic screening. Radiology. 2011; 258(1):98-105.

36. Osteras BH, Skaane P, Gullien R, Martinsen ACT. Average glandular dose in paired digital mammography and digital breast tomosynthesis acquisitions in a population based screening program: effects of measuring breast density, air kerma and beam quality. Phys Med Biol. 2018; 63(3):035006.

37. Byun J, Lee JE, Cha ES, Chung J, Kim JH. Visualization of Breast Microcalcifications on Digital Breast Tomosynthesis and 2-Dimensional Digital Mammography Using Specimens. Breast Cancer (Auckl). 2017; 11:1178223417703388.

38. Wei J, Chan HP, Hadjiiski LM, Helvie MA, Lu Y, Zhou C, Samala R. Multichannel response analysis on 2D projection views for detection of clustered microcalcifications in digital breast tomosynthesis. Med Phys. 2014; 41(4):041913.

39. Sung H, Ren J, Li J, Pfeiffer RM, Wang Y, Guida JL, Fang Y, Shi J, Zhang K, Li N, et al. Breast cancer risk factors and mammographic density among high-risk women in urban China. NPJ Breast Cancer. 2018; 4:3.

40. Oeffinger KC, Fontham ET, Etzioni R, Herzig A, Michaelson JS, Shih YC, Walter LC, Church TR, Flowers CR, LaMonte SJ, et al. Breast Cancer Screening for Women at Average Risk: 2015 Guideline Update From the American Cancer Society. JAMA. 2015; 314(15):1599-614.

41. Jin J. JAMA PATIENT PAGE. Breast Cancer Screening Guidelines in the United States. JAMA. 2015; 314(15):1658.

42. Ong JSL, Teh J, Saunders C, Bourke AG, Lizama C, Newton J, Phillips M, Taylor DB. Patient satisfaction with Radioguided Occult Lesion Localisation using iodine-125 seeds ('ROLLIS') versus conventional hookwire localisation. Eur J Surg Oncol. 2017; 43(12):2261-9.
